# Genistein attenuated gastric inflammation and apoptosis in *Helicobacter pylori*-induced gastropathy in rats

**DOI:** 10.1186/s12876-020-01555-x

**Published:** 2020-12-09

**Authors:** Prasong Siriviriyakul, Duangporn Werawatganon, Nisarat Phetnoo, Kanjana Somanawat, Tanittha Chatsuwan, Naruemon Klaikeaw, Maneerat Chayanupatkul

**Affiliations:** 1 Department of Physiology, Faculty of Medicine, Alternative and Complementary Medicine for Gastrointestinal and Liver Diseases Research Unit, Chulalongkorn University, Bangkok, 10330 Thailand; 2 Department of Microbiology, Faculty of Medicine, Alternative and Complementary Medicine for Gastrointestinal and Liver Diseases Research Unit, Chulalongkorn University, Bangkok, 10330 Thailand; 3 Department of Pathology, Faculty of Medicine, Alternative and Complementary Medicine for Gastrointestinal and Liver Diseases Research Unit, Chulalongkorn University, Bangkok, 10330 Thailand

**Keywords:** Genistein, *H. pylori*, Apoptosis, Gastric inflammation, Gastropathy

## Abstract

**Background:**

*Helicobacter pylori* (*H. pylori*) infection is a major cause of chronic gastritis, peptic ulcer diseases and cancer. Genistein (4′,5,7-trihydroxyisoflavone), a tyrosine-specific-protein kinase inhibitor, has been shown to exert an anti-inflammatory property**.** The aim of this study was to examine the treatment effects of genistein and its mechanisms in rats with *H. pylori* infection.

**Methods:**

Eighteen male Sprague-Dawley rats were divided into three groups (6 rats per group): (1) control group (Con); (2) *H. pylori* infected group (HP): the rats were inoculated with *H. pylori* (10^8−^ 10^10^ CFU/mL; 1 mL/rat*.*) for 3 consecutive days; and (3) HP + genistein group (HP + Gen): the rats were inoculated with *H. pylori* as above. Then, they were gavaged with genistein (16 mg/kg BW) for 14 days. Gastric tissue was used for the determination of nuclear factor (NF)-κB expression by immunohistochemistry (IHC), degree of apoptosis by the terminal deoxynucleotidyl transferasemediated dUTP nick-end labeling (TUNEL) reaction, and histopathology. Serum samples were used to measure the levels of tumor necrosis factor-alpha (TNF-α) and cytokine-induced neutrophil chemoattractant-1 (CINC-1).

**Results:**

Rats in the HP group had significantly higher levels of pro-inflammatory mediators, NF-κB expression and apoptotic cells when compared with the Con group, and these markers significantly decreased in HP + Gen group when compared with the HP group. The histopathology of HP group showed moderate gastric inflammation and many HP colonization. Gastric pathology in HP + Gen group demonstrated the attenuation of inflammatory cell infiltration and *H. pylori* colonization.

**Conclusion:**

Genistein exerted its gastroprotective effects through the reduction of pro-inflammatory mediators, nuclear receptor NF-κB expression and gastric mucosal apoptosis in rats with *H. pylori*-induced gastropathy.

## Background

*Helicobacter pylori* (*H. pylori*) is a spiral-shaped gram-negative bacterium and a major player in the development of gastroduodenal diseases. The prevalence of *H. pylori* infection ranges from 15 to 90% depending on the regions and the ethnicities [1]. Several studies demonstrated the association between *H. pylori* infection and numerous gastrointestinal diseases, such as chronic gastritis, peptic ulcer disease, gastric mucosa-associated lymphoid tissue (MALT) lymphoma, and gastric cancer [2–4]. To date, *H. pylori* infection remains the cause of major public health problem, especially in developing countries.

*H. pylori* causes gastric problems by first adhering to and colonizing the gastric mucosa. The bacteria then inject its virulent factors, particularly cagA protein, into the host epithelial cells using the type IV secretory system [3, 5]. These virulent factors affect host epithelial cell cytoskeletal rearrangements and activate NF-kB pathway, which subsequently induce IL-8 expression and release [6–8]. IL-8 is a potent chemotactic factor, thus attracting neutrophils and lymphocytes to the affected area. Consequently, these inflammatory cells release pro-inflammatory cytokines, such as tumor necrosis factor-alpha (TNF-α) and IL-6 leading to gastric mucosal injury and gastric epithelial cell apoptosis [9–11]. In response to TNF-α stimulation, gastric epithelial cells also release cytokine-induced neutrophil chemoattractant-1 (CINC-1) which works in concert with IL-8 in the attraction of neutrophils adding further insults to the injured mucosa [12].

The standard of care for the treatment of *H. pylori* infection includes the combination of acid suppression medication (proton pump inhibitor or vonoprazan) and 1–3 antibiotics for 7–14 days with the eradication rate ranging from 60 to 90% [13]. However, the resistance rates of the commonly used antibiotics can be more than 15% in some regions leading to unacceptable cure rates [14]. Furthermore, antibiotic use is associated with side effects which affects patient compliance, and may alter gut microbiota [15]. In light of these drawbacks, we strive to find an alternative and a more natural treatment for *H. pylori*-induced gastritis.

Genistein (4′,5,7-trihydroxyisoflavone) is a natural isoflavone found in soybeans and soy products. In vitro studies suggest that genistein is a specific inhibitor of tyrosine kinases [16], reduces inflammation through the inhibition of NF-kB activation and TNF-α production [17–19], and possesses anti-microbial properties to certain bacteria [20]. With these properties, we hypothesize that genistein may have a role in the treatment of *H. pylori* infection. Its treatment effect has also been implied by a case-control study by Ko KP and colleagues, which showed that genistein consumption was associated with the reduction in gastric cancer, particularly in *H. pylori*-infected subjects [21]. To the best of our knowledge, the effects of genistein on *H. pylori-*induced gastropathy in an experimental animal model have never been investigated. In the present study, we aimed to evaluate the anti-inflammatory effects of genistein, which may attenuate gastropathy through the suppression of nuclear receptor NF-κB expression, pro-inflammatory mediator production (i.e. TNF-α and CINC-1), and gastric epithelial cell apoptosis in rats with *H. pylori* infection.

## Methods

### Animal and chemical preparations

Eighteen male Sprague-Dawley rats (National Laboratory Animal Center, Mahidol University, Nakorn Pathom, Thailand) were used. The weight of rats ranged from 180 to 200 g at the start of the experiment. All experiments and procedures carried out on the animals have been approved by the Ethics Committee of the Faculty of Medicine, Chulalongkorn University, Bangkok, Thailand (IRB No. 8/57). Rats were housed in a controlled temperature room at 25 ± 1 °C and a 12 h light/dark cycle. All animals were allowed to acclimate to the new environment for 1 week prior to initiation of the experiment. Genistein (Batch number 0439241–61) were purchased from Cayman chemical company and dissolved in 0.1% DMSO.

### *H. pylori* strains and growth condition

*H. pylori* strains for all experiments were obtained from peptic ulcer patients at King Chulalongkorn Memorial Hospital. The bacteria were grown in brucella broth (pH 7.0) supplemented with 10% goat serum for 24 h at 37 °C in an automatic CO_2_-O_2_ incubator under microaerophillic conditions (85% N_2_, 10% CO_2_ and 5% O_2_).

### *H. pylori* inoculation model

The rats were inoculated with *H. pylori* using the method of Werawatganon et al. 2014 [22]. A 3-day treatment of streptomycin suspended in drinking water (5 mg/mL) was given to all rats in *H. pylori* groups prior to *H. pylori* inoculation. Then, they were inoculated with *H. pylori* suspension (10^8–10^ CFU/mL; 1 mL/rat) by intragastric tube twice a day, 4 h apart for 3 consecutive days. Two weeks later the rats were sacrificed**.**

Eighteen rats were randomly divided into three groups (six rats per group) as follows: [1] control group (Con), rats were treated with 0.1% DMSO (1 mL/rat) for 17 days [2]; *H. pylori*-infected group (HP), after *H. pylori* inoculation, rats were gavaged with 0.1% DMSO (1 mL/rat) as a vehicle control for 14 days; and, [3] HP + genistein group (HP + Gen), after *H. pylori* inoculation, rats were gavaged with genistein (16 mg/kg BW) dissolved in 0.1% DMSO for 14 days. At the end of the experiment, the rats were euthanized with overdose sodium pentobarbital (dose > 50 mg/kg) before collecting gastric tissue and blood samples.

### Measurement of TNF-α and CINC-1 levels in serum

Blood samples were obtained by cardiac puncture and centrifuged at 1500×g for 10 min. The supernatant was stored at − 80 °C until measurement. The serum concentrations of TNF-α and CINC-1 were measured by a rat TNF-α enzyme-linked immunosorbent assay and a rat CXCL1/CINC-1 Immunoassay (R and D systems, USA), respectively, according to the manufacturer’s protocols.

### NF-kB expression by immunohistochemistry (IHC) assay

Tissue sections were deparaffinized with xylene and ethanol for 10 min. The antigen was then retrieved with citrate buffer pH 6.0 in microwave for 13 min. Slides were incubated with 3% hydrogen peroxide (Merck, Hohenbrunn, Germany) for 5 min and with 3% normal horse serum (Gibco, Carlsbad, CA, USA) for 20 min to block endogenous peroxidase activity and nonspecific binding, respectively. Subsequently, gastric sections were incubated in a humidified chamber at room temperature for 1 h with a polyclonal antibody against NF-kβ (sc109; Santa Cruz Biotechnology, Santa Cruz, CA, USA) at a dilution of 1:100. Slides were then incubated with biotinylated anti-rabbit immunoglobulin (DAKO, Glostrup, Denmark) for 30 min. When the color development with diaminobenzidine (DAKO, Glostrup, Denmark) was seen, sections were counterstained with hematoxylin. Under light microscopy, positive cells were gastric epithelial cells with dark brown-stained nuclei. Under medium magnification (× 10 lens), one thousand gastric epithelial cells were manually counted for each rat to quantify the percentage of immune-positive cells. This procedure was performed by a pathologist who was blinded to the experiment. The percentage of immunoreactive cells was calculated as follows.

$$\mathrm{Percentage}\ \mathrm{of}\ \mathrm{immunoreactive}\ \mathrm{cells}\ \left(\%\right)=\left(\mathrm{number}\ \mathrm{of}\ \mathrm{immunoreactive}\ \mathrm{cells}\times 100\right)/1000.$$

### Gastric epithelial cells apoptosis by terminal deoxynucleotidyl transferase dUTP nick end labeling (TUNEL) assay

Apoptosis was determined by the presence of apoptotic nuclei in the gastric sections using fragment end labeling of DNA (Apoptosis detection kit, Chemicon, United States). First, the DNA fragments were bound to a peroxidase-conjugated antidigoxigenin antibody. After the development of a dark brown color by applying diaminobenzidine to the section, slides were counterstained with hematoxylin. Positive cells were those with dark brown nuclei under the light microscopy. To measure the degree of apoptosis, a total of 1000 gastric epithelial cells were counted in each rat to determine the numbers of the dark brown-stained cells. The data are presented as a percentage (%) of apoptotic cells using the following formula: the percentage of apoptotic cells (%) = (numbers of positive stained cells × 100)/1000.

### Gastric histopathology

A portion of gastric antral tissue was fixed in 10% formaldehyde solution. They were processed by a routine technique before paraffin embedding and sections were cut at 5 μm thickness. The sections were stained with hematoxylin-eosin (HE) and Giemsa staining methods. One experienced gastrointestinal pathologist examined all blinded samples by using light microscope following the updated Sydney System.^[46]^ All histopathological findings were recorded and graded by using the gastric inflammation score and bacterial colonization as follows: gastric inflammation score: 0: no infiltration of polymorphonuclear and mononuclear; 1: mild infiltration of polymorphonuclear and mononuclear; 2: moderate infiltration of polymorphonuclear and mononuclear; 3: severe infiltration of polymorphonuclear and mononuclear. *H. pylori* colonization score: 0: no bacteria detected; 1: few colonizations; 2: many colonizations.

### Statistical analysis

All data were presented as mean and standard deviation (SD). For comparison among all groups of animals, one-way analysis of variance (one-way ANOVA) comparisons were employed. Descriptive statistics were used for histological examination of the stomach. Differences were considered statistically significant at *P* < 0.05. The data were analyzed using SPSS software version 17.0 for Windows.

## Results

### Changes in levels of pro-inflammatory mediators, serum TNF-α and CINC-1

**Fig. 1:**
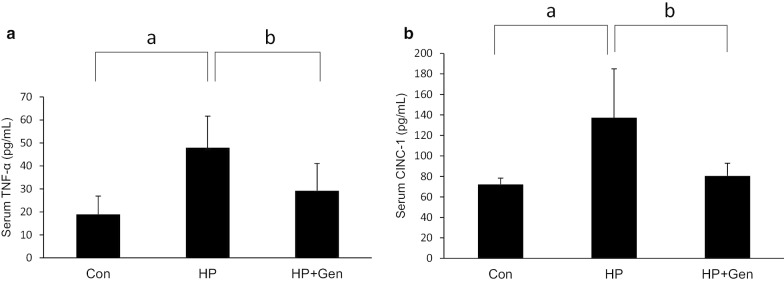
**a** Serum level of tumor necrosis factor-alpha and **b** cytokine-induced neutrophil chemoattractant-1 in all experimental groups (six rats each). Data are expressed as mean ± SD. (^a^*P* < 0.05 vs. Con group; ^b^*P* < 0.05 vs. HP group. Con: Control group; HP: *H. pylori* infection group; HP + Gen: *H. pylori* infection and genistein treatment group)

As shown in Fig. 1a and b, the HP group had significantly higher levels of serum TNF-α and CINC-1 when compared with the Con group (47.92 ± 13.74 pg/mL vs. 18.95 ± 7.94 pg/mL and 137.28 ± 47.67 pg/mL vs. 72.27 ± 6.07 pg/mL, respectively, *P* < 0.05). As expected, in the HP + Gen group, the serum levels of TNF-α and CINC-1 significantly decreased when compared with the HP group (29.24 ± 11.80 pg/mL vs. 47.92 ± 13.74 pg/mL and 80.48 ± 12.37 pg/mL vs. 137.28 ± 47.67 pg/m*L*, respectively*, P* < 0.05).

### NF-kB expression in gastric epithelial cells

**Fig. 2:**
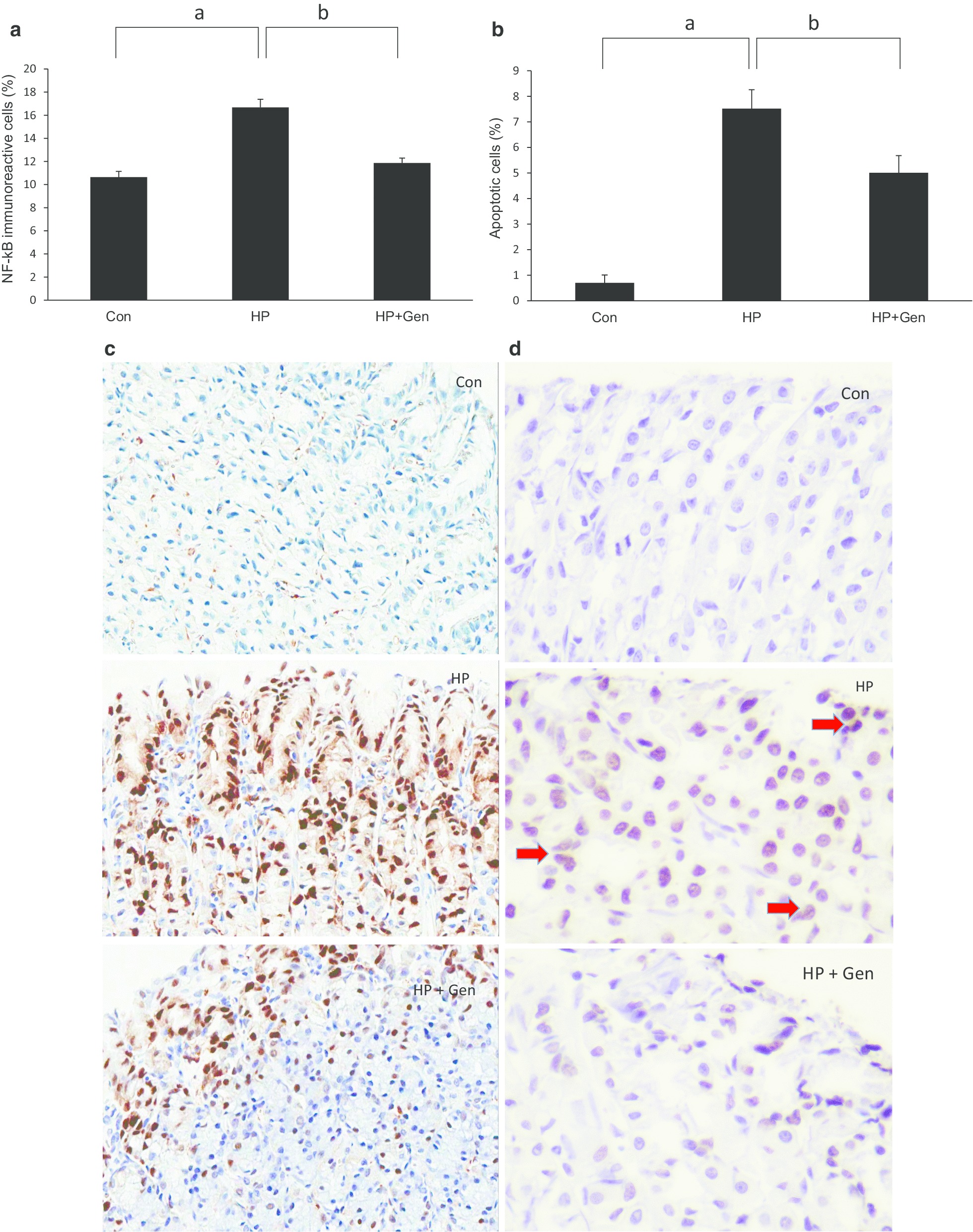
**a** The percentage of nuclear factor-kappaB immunoreactive cells (%) and **b** apoptotic cells (%) in all experimental groups (six rats each). Figure 2**c** were representative images of immunohistochemistry for NF-kappaB (× 40 magnification). Positive cells were gastric epithelial cells with brown-stained nuclei. Figure 2**d** were representative images of TUNEL stain (× 100 magnification). Arrows indicated gastric epithelial cells with brown-stained nuclei. Data are expressed as mean ± SD. **(**^a^*P* < 0.05 vs. Con group; ^b^*P* < 0.05 vs. HP group. Con: Control group; HP: *H. pylori* infection group; HP + Gen: *H. pylori* infection and genistein treatment group)

The percentage of NF-kB immunoreactive cells significantly increased in the HP group when compared with the Con group (16.67% ± 0.71% vs. 10.64% ± 0.50%, respectively, *p* < 0.05). The expression of NF-κB in gastric epithelial cells was attenuated by genistein administration in HP + Gen group when compared with the HP group (11.87% ± 0.42% vs. 16.67% ± 0.71%, respectively, *p <* 0.05). The average percentages of NF-κB positive gastric epithelial cells in all the groups are shown in Fig. 2a. The representative images of immunohistochemical study of NF-kB expression in each group are shown in Fig. 2c.

### Gastric epithelial cell apoptosis

As illustrated in Fig. 2b, the percentage of apoptotic cells significantly increased in the HP group when compared with the Con group (7.52% ± 0.74% vs. 0.70% ± 0.31%, respectively, *p* < 0.05). After treatment with genistein, the percentage of apoptotic cells significantly decreased in HP + Gen group when compared with the HP group (5.01% ± 0.67% vs. 7.52% ± 0.74%, respectively, *p* < 0.05). The representative images of gastric sections processed for apoptosis by the terminal deoxynucleotidyl transferase mediated dUTP nick-end labeling (TUNEL) reaction are shown in Fig. 2d.

### Histopathological changes

**Fig. 3:**
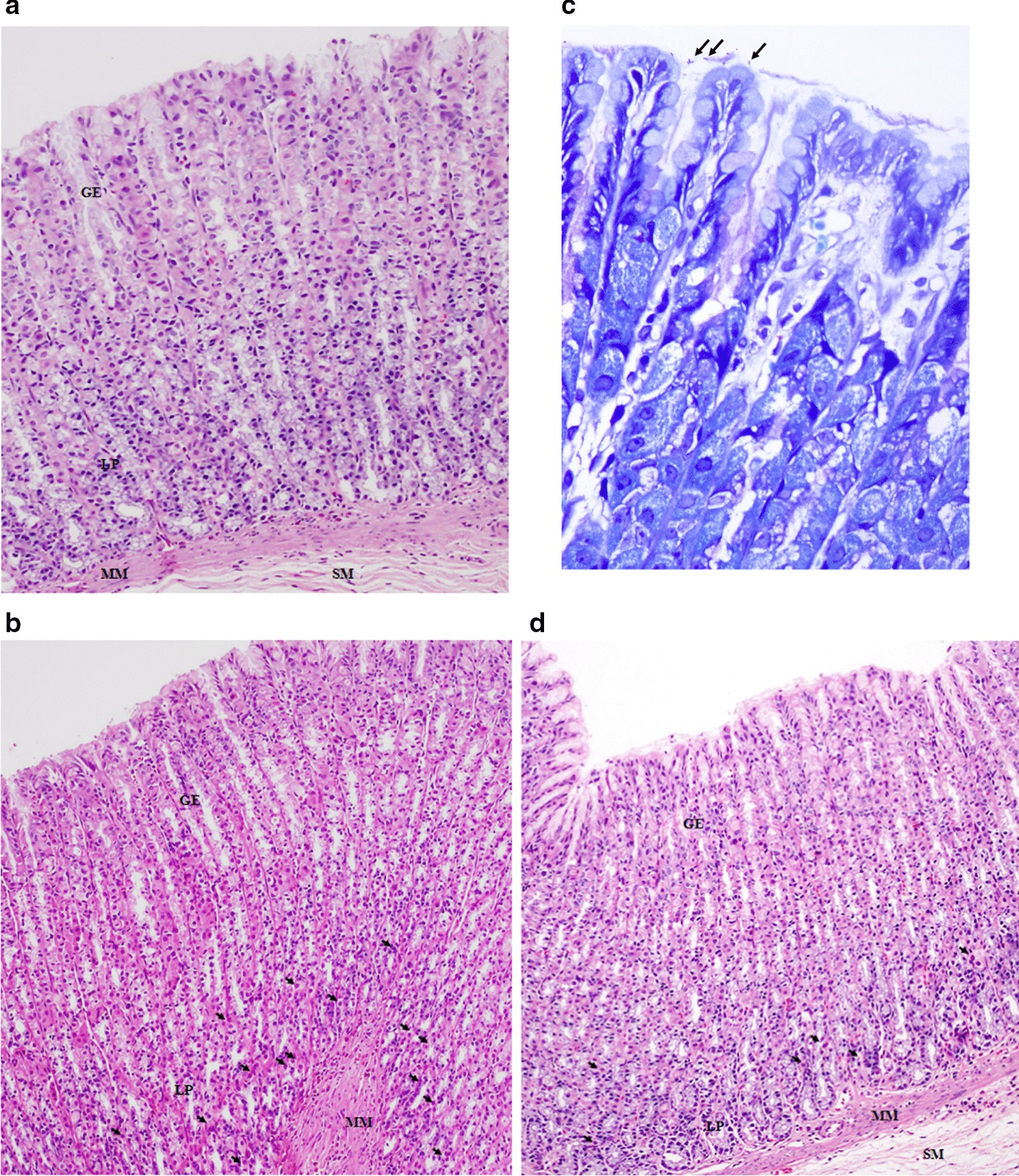
Gastric histopathology in rats with *H. pylori* infection. **a** Control group showed normal histopathology (H and E, × 10); **b** *H. pylori* infection group (H and E, × 10) with arrows indicating inflammatory cell infiltration in the lamina propria; **c** *H. pylori* infection group (Giemsa stain, × 100) with arrows indicating *H. pylori*; **d** *H. pylori* infection and genistein treatment group (H and E, × 10) with arrows indicating inflammatory cell infiltration in the lamina propria. GE: Gastric epithelium; LP: Lamina propria; MM: Muscularis mucosae; SM: Submucosa

As summarized in Table 1, the gastric histopathology was normal in the Con group, whereas in the HP group, the histopathology showed mild (*n* = 3) to moderate (*n* = 3) gastric inflammation. For *H. pylori* colonization, the HP group showed few (*n* = 3) and many (*n* = 3) colonization, while *H. pylori* colonization was not observed in the Con group. In HP + Gen group, histopathological changes were attenuated when compared to the HP group, especially in terms of inflammatory cell infiltration. Almost all of the gastric tissues in the HP + Gen group showed no infiltration of inflammatory cells (*n* = 5). Similarly*, H. pylori* colonization was reduced in the HP + Gen group (*n* = 5) (Fig. 3).

**Table 1:** Summary of the gastric inflammation and the bacterial colonization score

Group	N	Inflammation^**a**^				***H. pylori*** colonization^**b**^		
		No	Mild	Moderate	Severe	No	Few	Many
Con	6	6	–	–	–	6	–	–
HP	6	–	3	3	–	–	3	3
HP + Gen	6	5	1	–	–	5	1	–

^a^Gastric inflammation score: 0: no infiltration of polymorphonuclear and mononuclear; 1: mild infiltration of polymorphonuclear and mononuclear; 2: moderate infiltration of polymorphonuclear and mononuclear; 3: severe infiltration of polymorphonuclear and mononuclear

^b^*H. pylori* colonization score: 0: no bacteria detected; 1: few colonizations; 2: many colonizations

## Discussion

*H. pylori* infection is associated with the infiltration of polymorphonuclear and mononuclear cells in gastric mucosa. These inflammatory cells release several pro-inflammatory cytokines, reactive oxygen species, and proteolytic enzymes leading to mucosal damage [3]. Previous studies show that through the activation NF-kB, *H. pylori* induce the expression of IL-8 and TNF-α [9, 23, 24]. Another in vitro study also showed that the stimulation of gastric mucosal cell line with TNF-α led to increased oxidative stress and NF-kB activation with subsequent CINC-1 up-regulation [12]. The results from our study were in line with prior observations. We found that *H. pylori*-infected rats had higher serum levels of TNF-α and CINC-1, and increased expression of NF-kB in gastric epithelial cells as compared with the control group.

In vitro and in vivo studies suggest that *H. pylori* induces apoptosis mainly through the intrinsic pathway by means of increased Bak and Bax expression and cytochrome c release from mitochondria [11, 25]. In this study, we noted the increase in gastric epithelial cell apoptosis in *H. pylori*-infected rats. Similar to our results, Cover TL and colleagues performed an in vitro study using VacA positive *H. pylori* stain and AGS gastric epithelial cells. The authors found that VacA positive *H. pylori* stain could induce a higher level of apoptosis than VacA negative *H. pylori* stain, and purified VacA could dose-dependently induce apoptosis in AGS cells [26]. Similar findings were also seen in a human study by Moss SF and colleagues. They demonstrated a higher degree of gastric epithelial apoptosis in *H. pylori*-infected subjects with subsequent reduction in apoptosis after *H. pylori* eradication [27].

Geinstein, a major isoflavone found in soy-based products and a tyrosine-specific protein kinase inhibitor [16], has been shown to possess anti-neoplastic, anti-inflammatory, anti-oxidant, and anti-atherogenic properties [28–30]. Several in vitro studies demonstrated that genistein, as a tyrosine kinase inhibitor, could inhibit *H. pylori*-induced tyrosine-specific protein phosphorylation and IL-8 release from gastric epithelial cells suggesting that genistein could potentially be used in the treatment of *H. pylori* infection [31, 32]. In this study, we found that genistein administration reduced serum TNF-α and CINC-1, NF-kB expression, and gastric epithelial apoptosis in *H. pylori*-infected rats. As a result of these changes, gastric histopathology improved in *H. pylori*-infected rats that received genistein.

An in vitro model of lipopolysaccharide (LPS)-stimulated macrophages elucidated that genistein exerted its inhibitory effect on NK-kB activation through the reduction in lipid peroxidation and the increase in glutathione and anti-oxidant enzyme activities [19]. Additionally, genistein could suppress NK-kB activation in LPS-stimulated macrophages through adenosine monophosphate-activated protein kinase (AMPK) pathway, thereby reducing TNF-α and IL-6 production [17]. These mechanism likely explained the reduction in NF-kB expression and pro-inflammatory cytokine production after genistein administration in *H. pylori*-infected rats in our study.

In the present study, gastric pathology in the majority of genistein-treated rats showed the disappearance of neutrophil infiltration in conjunction with the decrease in *H. pylori* colonization. Similar findings were reported in a pathology study in humans [33]. The authors showed that neutrophil infiltration diminished in patients with *H. pylori* eradication and the presence of residual neutrophils predicted relapses [33]. Takekawa S and colleagues demonstrated that genistein could reduce the production of thiobarbituric acid reactive substances (TBARS), TNF-α and CINC-1 in gastric mucosa in a rat model of stress-induced mucosal injury [34]. The reduction in CINC-1, a potent neutrophil chemotactic factor, was likely responsible for the disappearance of neutrophil infiltration in a genistein-treated group. Likewise, genistein has been shown to reduce the levels of malondialdehyde and TNF-α in a rat model of indomethacin-induced gastropathy [35, 36]. The decline in inflammatory responses and oxidative stress also ameliorated gastric mucosal injury and gastric epithelial cell apoptosis in *H. pylori*-infected rats that received genistein.

A prior in vitro study suggested that by binding to α5β1-integrins on gastric epithelial cell surface, *H. pylori* could induce tyrosine phosphorylation of proteins that were responsible for bacterial adherence to gastric epithelial cells. The adherence of *H. pylori* to gastric cells could be inhibited by tyrosine kinase inhibitors, such as genistein [37]. This mechanism likely explained the decrease in *H. pylori* colonization in genistein-treated rats in our study, since *H. pylori* adherence to gastric mucosal cells is paramount in the pathogenesis of *H. pylori* infection. Although we do not have prior evidence to suggest that genistein has a bactericidal effect on *H. pylori*, in vitro studies using other bacteria supported that genistein might at least be a bacteriostatic compound [38].

## Conclusions

*H. pylori* infection is associated with elevated levels of pro-inflammatory mediators (TNF-α, and CINC-1), increased neutrophil infiltration in gastric mucosa with *H. pylori* colonization, and gastric epithelial cell apoptosis. Genistein exerted its gastroprotective effects in rats with *H. pylori*-induced gastropathy through the reduction in NF-kB activation, pro-inflammatory cytokine production, and gastric apoptotic cell death. Clinical studies are needed to confirm the therapeutic effects of genistein in human.

## Data Availability

The datasets used and/or analysed during the current study are available from the corresponding author on reasonable request.

## Abbreviations

*H. pylori*: *Helicobacter pylori*
TNF-α: Tumor necrosis factor-alpha
CINC-1: Cytokine-induced neutrophil chemoattractant-1
NF-kB: Nuclear factor-kappaB
IL-1: Interleukin-1
IL-6: Interleukin-6
IL-8: Interleukin-8
IL-1β: Interleukin-1beta
EGF: Epidermal growth factor
LPSs: Lipopolysaccharide
TUNEL: Terminal deoxynucleotidyl transferase mediated dUTP nick-end labeling
IHC: Immunohistochemistry
ELISA: Enzyme-linked immunosorbent assay
HE: Hematoxylin-eosin
